# Identification of crucial modules and genes associated with backfat tissue development by WGCNA in Ningxiang pigs

**DOI:** 10.3389/fgene.2023.1234757

**Published:** 2023-08-17

**Authors:** Chen Chen, Huibo Ren, Huali Li, Yuan Deng, Qingming Cui, Ji Zhu, Siyang Zhang, Jine Yu, Huiming Wang, Xiaodan Yu, Shiliu Yang, Xionggui Hu, Yinglin Peng

**Affiliations:** ^1^ Department of Pig Breeding, Key Laboratory of Conservation and Genetic Analysis of Indigenous Pigs, Hunan Institute of Animal and Veterinary Science, Changsha, China; ^2^ Hunan Liushahe Ecological Animal Husbandry Co, Ltd., Changsha, China; ^3^ Department of Animal Genetics and Breeding, College of Animal Science and Technology, Hunan Agricultural University, Changsha, China

**Keywords:** Ningxiang pig, backfat tissue, different developmental stage, WGCNA, hub gene, lipid metabolism

## Abstract

Fat deposition is an economically important trait in pigs. Ningxiang pig, one of the four famous indigenous breeds in China, is characterized by high fat content. The underlying gene expression pattern in different developmental periods of backfat tissue remains unclear, and the purpose of this investigation is to explore the potential molecular regulators of backfat tissue development in Ningxiang pigs. Backfat tissue (three samples for each stage) was initially collected from different developmental stages (60, 120, 180, 240, 300, and 360 days after birth), and histological analysis and RNA sequencing (RNA-seq) were then conducted. Fragments per kilobase of transcript per million (FPKM) method was used to qualify gene expressions, and differentially expressed genes (DEGs) were identified. Furthermore, strongly co-expressed genes in modules, which were named by color, were clustered by Weighted gene co-expression network analysis (WGCNA) based on dynamic tree cutting algorithm. Gene ontology (GO) and kyoto encyclopedia of genes and genomes (KEGG) enrichment were subsequently implemented, and hub genes were described in each module. Finally, QPCR analysis was employed to validate RNA-seq data. The results showed that adipocyte area increased and adipocyte number decreased with development of backfat tissue. A total of 1,024 DEGs were identified in five comparison groups (120 days vs. 60 days, 180 days vs. 120 days, 240 days vs. 180 days, 300 days vs. 240 days, and 360 days vs. 300 days). The turquoise, red, pink, paleturquoise, darkorange, and darkgreen module had the highest correlation coefficient with 60, 120, 180, 240, 300, and 360 days developmental stage, while the tan, black and turquoise module had strong relationship with backfat thickness, adipocyte area, and adipocyte number, respectively. Thirteen hub genes (*ACSL1*, *ACOX1*, *FN1*, *DCN*, *CHST13*, *COL1A1*, *COL1A2*, *COL6A3*, *COL5A1*, *COL14A1*, *OAZ3*, *DNM1*, and *SELP*) were recognized. *ACSL1* and *ACOX1* might perform function in the early developmental stage of backfat tissue (60 days), and *FN1*, *DCN*, *COL1A1*, *COL1A2*, *COL5A1*, *COL6A3*, and *COL14A1* have unignorable position in backfat tissue around 120 days developmental stage. Besides, hub genes *SELP* and *DNM1* in modules significantly associated with backfat thickness and adipocyte area might be involved in the process of backfat tissue development. These findings contribute to understand the integrated mechanism underlying backfat tissue development and promote the progress of genetic improvement in Ningxiang pigs.

## Introduction

Pigs are not only a major source of meat worldwide, but also have been used in biomedical studies because of the similarity to human in physiology (Miao et al., 2018). Ningxiang pig, one of the four famous indigenous pig breeds in China, is native to Hunan province and is characterized by high fat content, delicious meat, strong adaptability to the local environment, and stronger disease resistance (Li et al., 2021). Adipose tissue secretes a variety of proteins that impact a number of physiological and metabolic processes (Hausman et al., 2007). Adipose tissue in pigs is an important trait, which influences meat quality and fattening efficiency (Stachowiak et al., 2016), and backfat deposition greatly influences porcine growth performance, carcass, meat production and final farming profit (Davoli et al., 2018).

Weighted gene co-expression network analysis (WGCNA) is a bioinformatics algorithm method that has been widely utilized to explore highly correlated gene clusters related to biological traits (Wang et al., 2022; Wu et al., 2022; Xu et al., 2022). Instead of focusing on a single gene, WGCNA intends to extract hub genes from co-expression networks to preferably investigate the biological regulations. Recently, WGCNA method was popularly applied, and mounting genes were found to participate in various lipid related processes, including obesity, adipogenic differentiation of stem cell, polyunsaturated fatty acid (Han et al., 2020; Liu et al., 2021; Xiao et al., 2022; Zhang et al., 2022). Although the study on gene profiles in subcutaneous adipose tissue at four developmental stages in Ningxiang pigs has been reported (Gong et al., 2021), the underlying molecular mechanism of subcutaneous adipose tissue development in different periods is still unclear.

In view of the important role of adipose tissue, the present study collected backfat tissue across six postnatal developmental stages (60, 120, 180, 240, 300, and 360 days after birth, hereafter referred to as 60, 120, 180, 240, 300, and 360 days, respectively) in Ningxiang pigs. These time points cover major morphological and physiological changes in pig growth and development due to the fact that backfat tissue development in pigs is varying according to their age (Mersmann et al., 1973; Hood and Allen, 1977).

Here, backfat tissue was subjected to histological analysis and RNA-sequencing (RNA-seq). The genes profiles in backfat tissue from six developmental stages were investigated, and differentially expressed genes (DEGs) in five comparison groups were identified. Furthermore, WGCNA was performed to analyze the gene expression profile, and the key modules and hub genes with strong correlation with developmental stages and traits were explored, respectively. In addition, RNA-seq result was validated by quantitative real-time polymerase chain reaction (QPCR) experiment. These findings unprecedentedly depicted the gene co-expression network for backfat tissue development at each period in Ningxiang pigs, which not only helps to understand the integrated mechanism underlying lipid metabolism, but also promotes the progress of genetic improvement in pigs.

## Materials and methods

### Experimental animal and sample preparation

Eighteen castrated male Ningxiang pigs in six developmental stages (60, 120, 180, 240, 300, and 360 days) were used in this study. Six groups of pigs were half-sibs, and the three samples in each developmental stage were full-sibs. All the experimental pigs were reared under the standard environmental conditions in Hunan Liushahe Ecological Animal Husbandry Co., Ltd. Three healthy individuals with similar body weight were selected for slaughtering in each developmental stage. The carcass was split longitudinally after removing the head, feet, tail, and viscera, except for the suet and kidneys, and then the left side of carcass was hung upside down. The midline backfat thickness at the position of the thickest point in shoulder, last rib and lumbosacral junction was separately measured, and the average value was calculated. Furthermore, the backfat tissue at the position of the thickest point in shoulder were collected within 30 min after slaughter, and each sample was divided into two parts. One part was fixed in 10% paraformaldehyde for histological analysis, and the other part was immediately frozen in liquid nitrogen and stored in −80°C refrigerator for RNA extraction.

### Histological analysis of backfat tissue

The histological analysis of backfat tissue was measured by hematoxylin and eosin (HE) staining. Briefly, the paraformaldehyde-fixed backfat samples were subjected to dehydration and embedding in paraffin. Three serial tissue sections of each sample were obtained using Leica cryostat (RM 2016, Germany) and then stained with hematoxylin/eosin. Adipocyte area and adipocyte number were calculated according to four randomly selected fields from each section using Caseviewer software. The sections were viewed at ×200 magnification using 3D digital scanner (Pannoramic 250, Hungary).

### RNA exaction, library construction and data processing

Total RNA was isolated using Trizol reagent (Invitrogen, United States) following the manufacturer’s protocol. The purity and integrity of total RNA for each sample were assessed using ND-1000 (NanoDrop, United States) and bioanalyzer 2100 (Agilent, United States) with RIN number >7.0, and confirmed by electrophoresis with denaturing agarose gel. And then mRNA library was constructed. In brief, approximately 5 μg of total RNA was used to eliminate ribosomal RNA according to the Ribo-Zero™ rRNA removal Kit (Illumina, United States), and the ribo-minus RNA was fragmented into small pieces. Finally, the 2 × 150 bp paired-end sequencing (PE150) was executed on an Illumina Novaseq™ 6000 (LC-Bio Technology Co., Ltd., China). Furthermore, Cutadapt (V1.9, default) (Martin, 2011) was initially applied to obtain valid data by removing the reads that contained adapter contamination, low quality bases (the number of bases with quality score ≤10 accounts for more than 20% of reads) and undetermined bases (the number of bases with undetermined information accounts for more than 5% of reads). Then sequence quality was verified using FastQC (V0.11.9, default) (Brown et al., 2017). Hisat2 software (V2.0.4, default) (Kim et al., 2019) was used to map valid reads to the *Sus scrofa* reference genome (V11.1, Ensembl V96), and the mapped reads were assembled using StringTie (V1.3.4, default) (Pertea et al., 2015).

### Identification and functional enrichment of DEGs

The expression levels of genes were estimated by calculating fragments per kilobase of transcript per million (FPKM) (Trapnell et al., 2010) through StringTie. The DEGs were selected with | log2 (fold change) | ≥ 1 and FDR adjusted p-value (q value) (Benjamini-Hochberg method) ≤ 0.05 by R package edgeR (Robinson et al., 2010). The volcano plot was drawn using ggplot2 package (Ito and Murphy, 2013), while the heatmap was drawn by pheatmap package (Hu, 2021). To assess the potential biological functions of DEGs, gene ontology (GO) (Ashburner et al., 2000) and kyoto encyclopedia of genes and genomes (KEGG) (Kanehisa et al., 2012) enrichment analysis were performed by DAVID software (https://david.ncifcrf.gov/). GO terms and KEGG pathways with p-value ≤0.05 were considered significantly enriched.

### WGCNA

Co-expression analysis was performed using WGCNA package in R (Langfelder and Horvath, 2008) under the guidelines of the published tutorials. Genes with FPKM <1 in all samples were filtered. Hierarchical clustering of the eighteen samples was conducted based on Euclidean distance computed on gene expression data. Network topology analysis ensured a scale-free topology network with the defined soft-thresholding power of 9. A total of 41 modules were identified based on the dynamic tree cutting algorithm with the parameters of minModuleSize at 30 and mergeCutHeight at 0.25. For each module, the eigengene (the first component expression of genes in module) was determined, and the correlations of eigengenes with backfat thickness, adipocyte area, and adipocyte number were then subsequently calculated. Genes with high connectivity in the respective modules were considered hub genes. The co-expression relationships in modules were analyzed and visualized by Cytoscape (V3.8.2) (Shannon et al., 2003).

### QPCR analysis

To validate the gene expression levels, total RNA was extracted from backfat tissue in six developmental stages using Trizol reagent (Invitrogen, United States). cDNA was synthesized using RevertAid™ first strand cDNA synthesis kit (K1622, Fermentas) according to the manufacturer’s instructions. QPCR analysis was performed using SYBR Green Supermix (Biomed) on CFX96 machine (Bio-Rad, United States). Porcine *β-actin* was used as endogenous control. Each QPCR reaction was performed in triplicate, and the relative expression level of gene was calculated using the 2^−ΔΔCT^ method. The sequences of QPCR primers were listed in Supplementary Table S1.

## Results

### Histological analysis of backfat tissue

**FIGURE 1:**
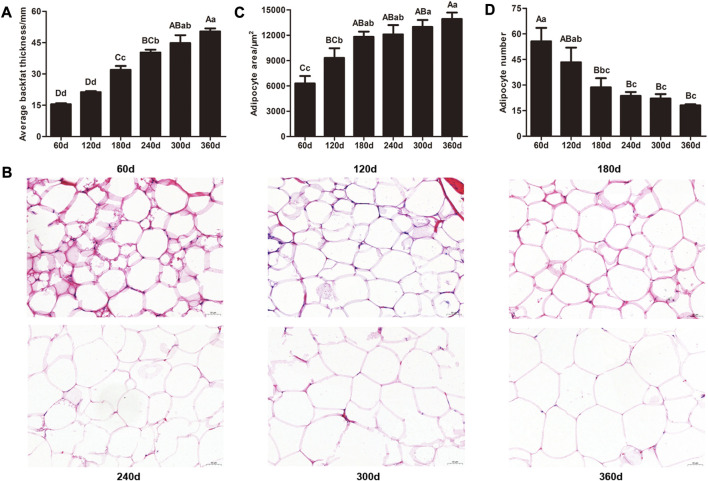
Histological analysis of backfat tissue from the six developmental stages in Ningxiang pigs. **(A)** Average backfat thickness of experimental pigs. **(B)** Representative images of HE staining of backfat tissue (magnification: ×200, scale bar = 50 μm). **(C)** Adipocyte area and **(D)** adipocyte number of backfat tissue. Statistical analysis of the data from backfat thickness, adipocyte area, and adipocyte number were performed by one-way analysis of variance program with SPSS 20.0 software. All results were expressed as mean values and standard error. Different lowercase letters above columns indicated statistical differences (*p* ≤ 0.05), and values with different uppercase letters were significantly different (*p* ≤ 0.01).

With development of backfat tissue in Ningxiang pigs, the average backfat thickness in 60 and 120 days were significantly lower than that in other stages (*p* < 0.01), and there were remarkable differences in the average backfat thickness between 180 and 240 days (*p* < 0.05), 180 and 300 days (*p* < 0.01), 180 and 360 days (*p* < 0.01), 240 and 360 days (*p* < 0.01), respectively (Figure 1A). Additionally, obvious differences in adipocyte phenotype were examined by HE staining (Figure 1B). Adipocyte area gradually increased, and adipocyte number showed a progressive downward trend. Adipocyte area in 60 days was notably smaller than that in other stages, and 300 days (*p* < 0.05) and 360 days (*p* < 0.01) had larger adipocyte area than 120 days. Meanwhile, the adipocyte number in 60 days was markedly higher (*p* < 0.01) that than in other stages except for 120days, and the adipocyte numbers in 240, 300, and 360 days were lower (*p* < 0.05) compared with that in 120 days. These results displayed the intelligible features during development of backfat tissue in Ningxiang pigs.

### Overview of RNA-seq data

A total of eighteen libraries were constructed using backfat tissue from the developmental stage of 60, 120, 180, 240, 300, and 360 days. We obtained 89,249,470–97,751,248 raw reads and 81,225,086–90,086,352 valid reads with Q30 ratio of 97.56%–97.99% and GC content of 45.50%–51.50%. Moreover, 87.35%–91.85% of valid reads was mapped to the *Sus scrofa* reference genome. After assembly, 56,294–57,783 transcripts and 23,416–24,057 genes were obtained (Supplementary Table S2). In addition, a total of 27,883 genes were identified from the eighteen libraries, including 19,755 known genes and 8,128 novel genes.

### Identification of DEGs

**FIGURE 2:**
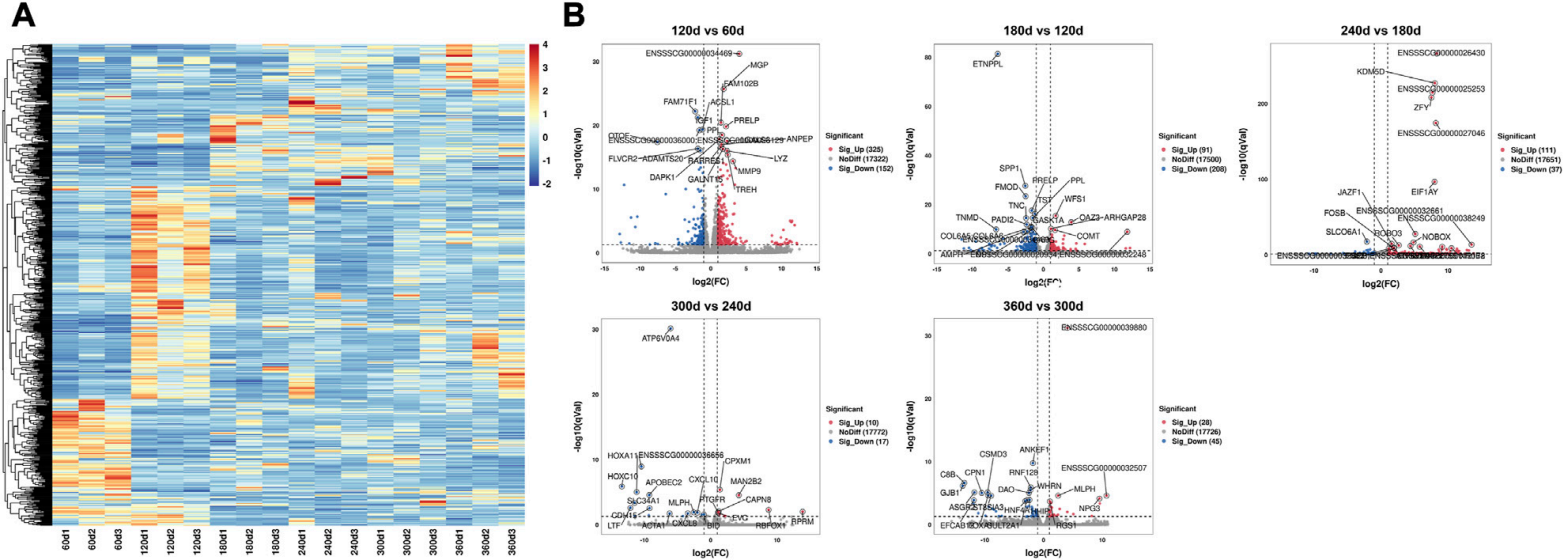
The heatmap and volcano plot of 1,024 DEGs in different developmental stages. **(A)** The heatmap of DEGs in six developmental stages. Each row indicated a gene, and each column indicated a sample. **(B)** The volcano plot of DEGs in 120 days vs. 60 days, 180 days vs. 120 days, 240 days vs. 180 days, 300 days vs. 240 days, and 360 days vs. 300 days comparison groups. The X-axis represented log2 value of fold change, and the Y-axis represented–log10 value of q value for each gene. The red dots demonstrated the significantly upregulated genes while the blue dots demonstrated the significantly downregulated genes, and the grey dots demonstrated the genes with no significant differential expression. The top 20 DEGs with significant difference were noted.

Five comparison groups (120 days vs. 60days, 180 days vs. 120days, 240 days vs. 180days, 300 days vs. 240 days, and 360 days vs. 300 days) were established. A total of 1,024 DEGs were recognized (Table 1), and the DEGs among these groups were visualized as heatmap (Figure 2A) and volcano plot (Figure 2B), respectively. The detailed information about these DEGs was documented in Supplementary Table S3.

**TABLE 1:** The number of DEGs in five comparison groups.

Comparison group	Number of DEGs		
	Upregulation	Downregulation	Total
120 days vs. 60 days	325	152	477
180 days vs. 120 days	91	208	299
240 days vs. 180 days	111	37	148
300 days vs. 240 days	10	17	27
360 days vs. 300 days	28	45	73
Total	565	459	1,024

### WGCNA

**FIGURE 3:**
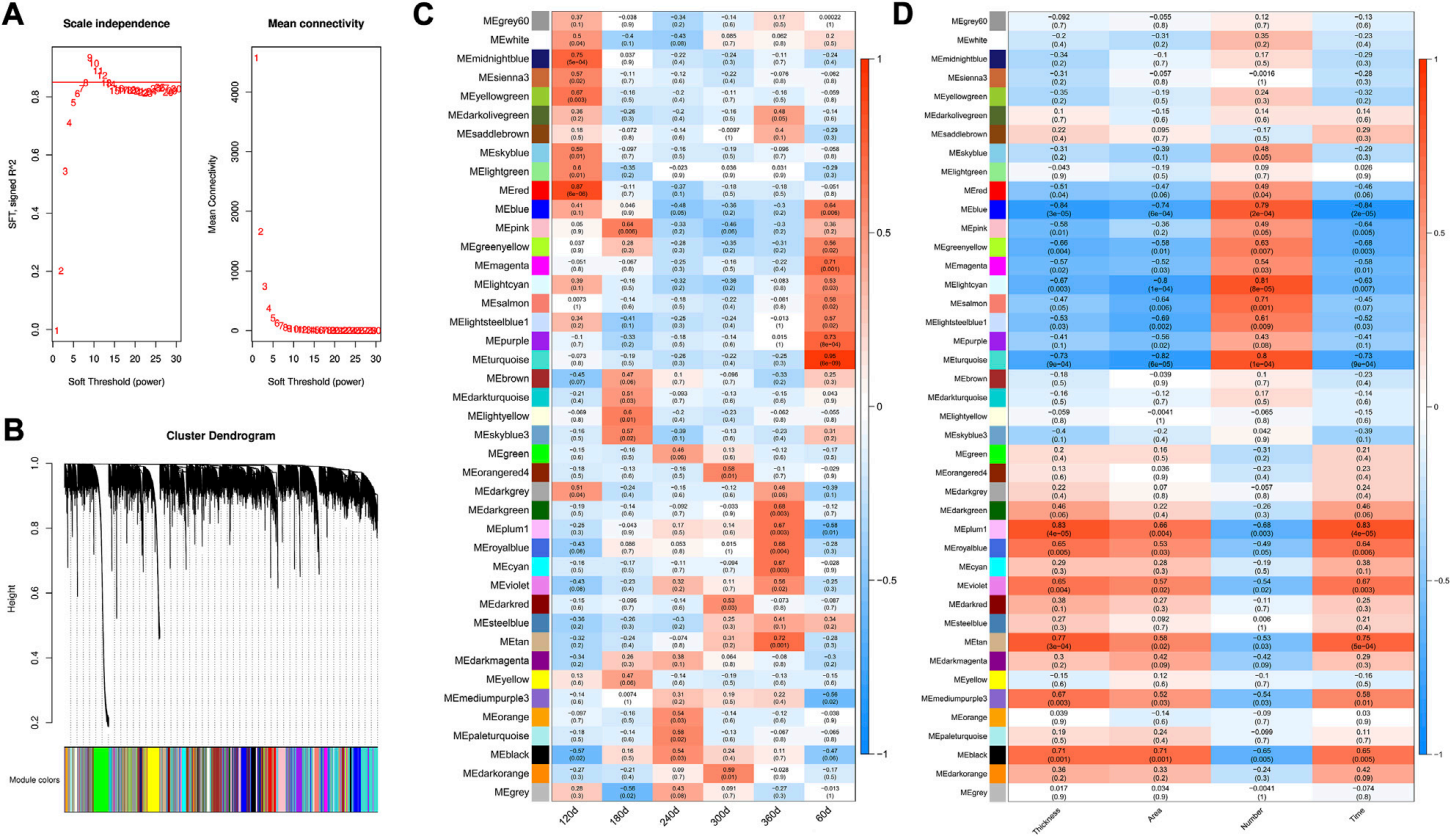
Weighted gene co-expression network construction. **(A)** Determination of best soft-threshold β for WGCNA. The red line corresponding to 0.85. Nine is selected based on the consideration of both scale independence and mean connectivity. SFT means scale free topology. **(B)** Clustering tree (dendrogram) defined by WGCNA representing the co-expression modules. Branches of the dendrogram correspond to gene modules labeled with different colors **(C)** Heatmap of correlation coefficient between genes modules and developmental stages. **(D)** Heatmap of correlation coefficient between genes modules and traits. Thickness: backfat thickness; Area: adipocyte area; Number: adipocyte number. Red indicated a strong positive association, and blue indicated a strong negative correlation. The numbers in brackets represent the p-value between gene module and developmental stage/trait.

A cut-off of R^2^ = 0.85 was utilized to select the soft-threshold β, and β = 9 was selected for network construction (Figure 3A). Strongly co-expressed genes in modules were clustered with different colors, while the genes not clustered were grouped into the grey module (Figure 3B). The detailed information about modules was shown in Supplementary Table S4. The correlation in module-stage relationship was displayed as a heatmap (Figure 3C). Among these modules, turquoise (0.95, p = 6e-09), red (0.87, p = 6e-06), pink (0.64, *p* = 0.006), paleturquoise (0.58, *p* = 0.02), darkorange (0.59, *p* = 0.01), and darkgreen (0.68, *p* = 0.003) module had the highest correlation coefficient with 60, 120, 180, 240, 300, and 360 days, respectively. Meanwhile, the correlation of modules with traits was presented in Figure 3D, and tan (0.77, p = 3e-04), black (0.71, *p* = 0.001) and turquoise (0.80, p = 1e-04) had strong correlation with backfat thickness, adipocyte area, and adipocyte number, respectively.

### Functional enrichment of genes in modules

**FIGURE 4:**
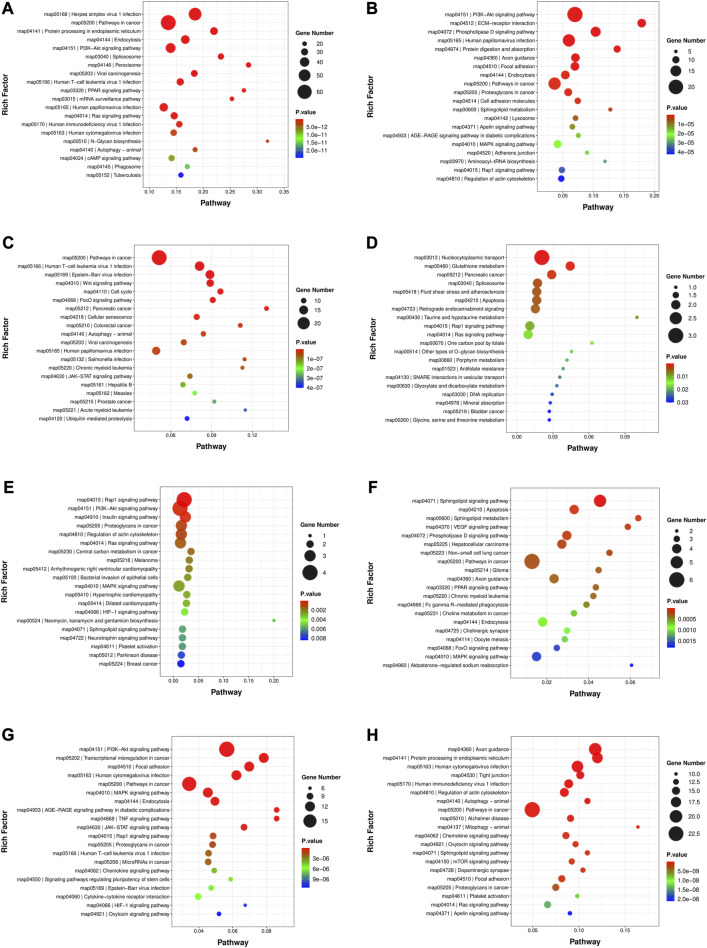
The top 20 significantly enriched KEGG pathways in developmental stage related and trait related modules. **(A)** Turquoise module. **(B)** Red module. **(C)** Pink module. **(D)** Paleturquoise module. **(E)** Darkorange module. **(F)** Darkgreen module. **(G)** Tan module. **(H)** Black module.

The GO analysis and KEGG enrichment were performed for the genes in the turquoise, red, pink, paleturquoise, darkorange and darkgreen modules in module-stage relationship, respectively. The result of GO analysis was illustrated in Figure S1; Supplementary Table S5. The comprehensive content about the KEGG enriched pathways was exhibited in Supplementary Table S6, and the top 20 significantly enriched pathways were shown in Figures 4A–F, respectively. The turquoise module, which was related to 60 days, was enriched with 306 pathways, of which 245 pathways were noticeably enriched, such as fatty acid degradation, glycerolipid metabolism, glycerophospholipid metabolism, fatty acid elongation, biosynthesis of unsaturated fatty acids, ether lipid metabolism, alpha-linolenic acid metabolism, sphingolipid signaling pathway, non-alcoholic fatty liver disease, fat digestion and absorption, adipocytokine signaling pathway, regulation of lipolysis in adipocyte, bile secretion, and cholesterol metabolism. A total of 121 terms were distinctly enriched in red module, and the KEGG pathways included glycerolipid metabolism, glycerophospholipid metabolism, ether lipid metabolism, sphingolipid metabolism, arachidonic acid metabolism, and regulation of lipolysis in adipocyte. The pink module correlated to 180 days were enriched in 152 significant pathways. For example, sphingolipid metabolism, sphingolipid signaling pathway, non-alcoholic fatty liver disease, and adipocytokine signaling pathway were all connected with lipid metabolism. There were 60 KEGG pathways in the paleturquoise module, of which 30 were remarkably enriched, including steroid hormone biosynthesis and arachidonic acid metabolism. The darkorange module correlated to 300 days was strikingly enriched in 50 terms, of which fatty acid biosynthesis, sphingolipid metabolism, steroid hormone biosynthesis, and sphingolipid signaling pathway were detected. The darkgreen module was enriched with 127 pathways, of which 50 pathways were significantly enriched, such as sphingolipid metabolism, fatty acid degradation, fatty acid biosynthesis, primary bile acid biosynthesis, steroid biosynthesis, sphingolipid signaling pathway, and adipocytokine signaling pathway.

Furthermore, the GO analysis (Supplementary Figure S1; Supplementary Table S5) and KEGG enrichment of genes in module-trait relationship were also carried out. As exhibited in Figure 4; Supplementary Table S7, in the notably KEGG enriched pathways related to lipid metabolism, the tan module (Figure 4G) was involved in sphingolipid signaling pathway, regulation of lipolysis in adipocytes and adipocytokine signaling pathway; the black module (Figure 4H) were associated with glycerophospholipid metabolism, arachidonic acid metabolism, fatty acid degradation, glycerolipid metabolism, fatty acid elongation, adipocytokine signaling pathway, and regulation of lipolysis in adipocytes; the turquoise module (Figure 4A) was the same as that in module-stage relationship.

### Network analysis and hub genes identification

**FIGURE 5:**
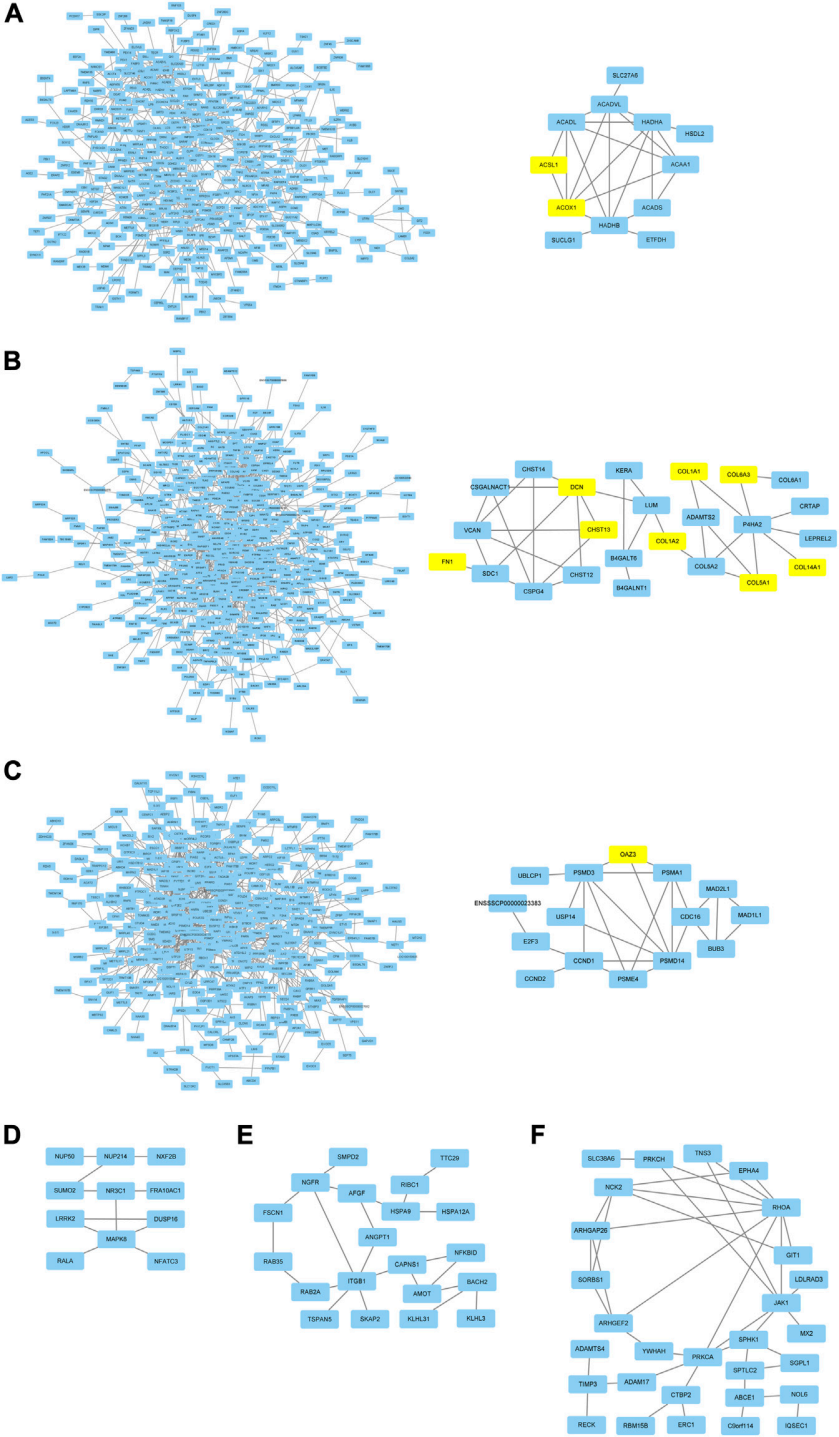
Gene co-expression networks and hub genes in developmental stage related modules. **(A)** Network (left) and hub genes (right) in turquoise module. **(B)** Network (left) and hub genes (right) in red module. **(C)** Network (left) and hub genes (right) in pink module. **(D)** hub genes in paleturquoise module. **(E)** hub genes in darkorange module. **(F)** hub genes in darkgreen module. The yellow rectangles represented DEGs.

**FIGURE 6:**
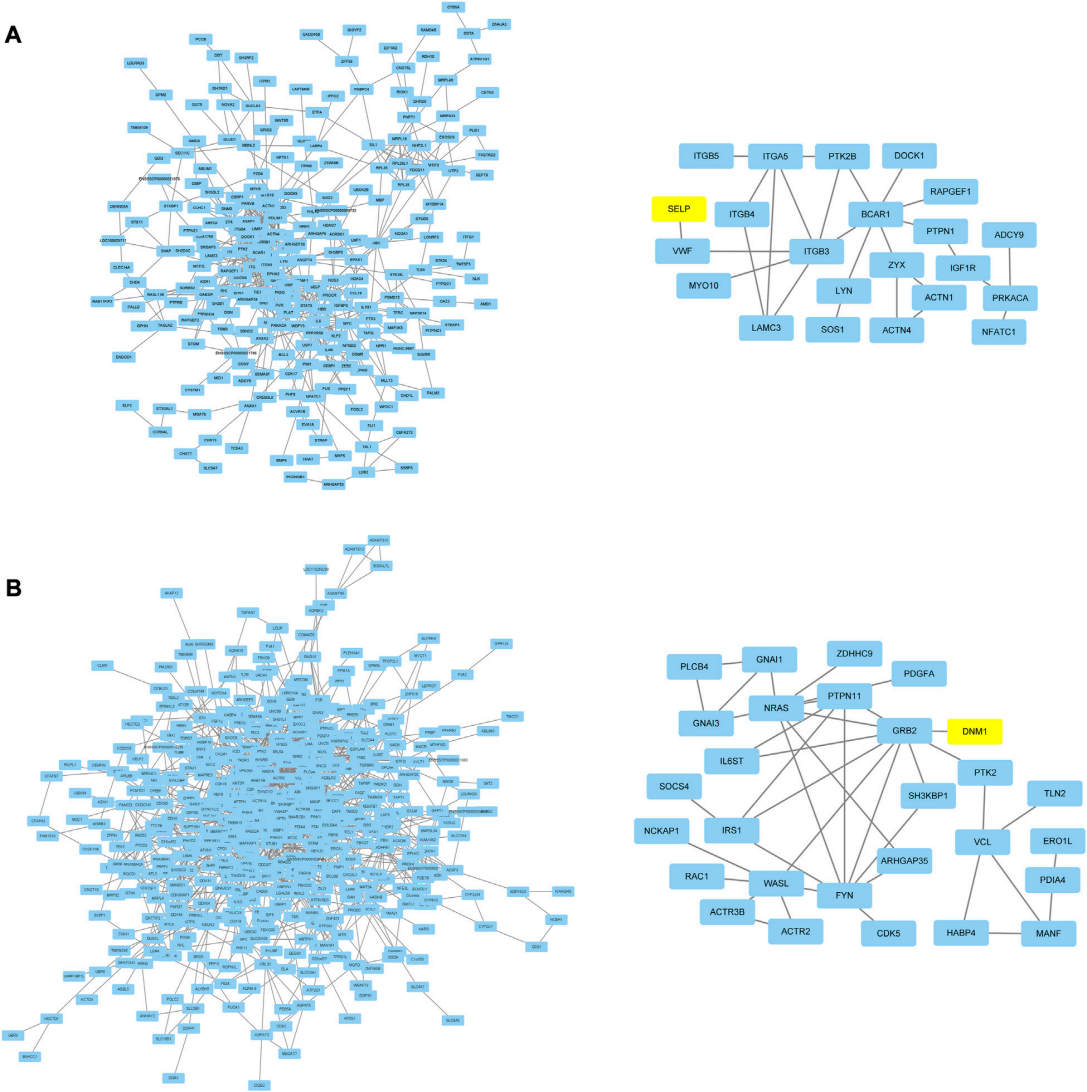
Gene co-expression networks and hub genes in trait related modules. **(A)** Network (left) and hub genes (right) in tan module. **(B)** Network (left) and hub genes (right) in black module. The yellow rectangles represented DEGs.

In order to research the interaction of genes in each module based on module-stage and module-trait relationship, the gene co-expression network was constructed by cytoscape software. And the connectivity between genes in each module was listed in Supplementary Table S8. The gene co-expression network with 364 nodes and 741 edges, 470 nodes and 1,100 edges, 380 nodes and 1,051 edges, 27 nodes and 21 edges, 40 nodes and 35 edges, and 54 nodes and 56 edges in turquoise, red, pink, paleturquoise, darkorange, and darkgreen module, respectively. In addition, the gene co-expression network with 238 nodes and 557 edges and 498 nodes and 1,427 edges in tan and black module. After removing the isolated nodes and node pairs, the gene co-expression network in module was visualized. The top 10% connectivity genes in turquoise, red and pink module and all connectivity genes in paleturquoise, darkorange and darkgreen module with the greatest number of edges were considered as hub genes (Figures 5A–F). And the top 10% connectivity genes in tan and black module with the greatest number of edges were also regarded as hub genes (Figure 6).

### QPCR validation of DEGs and hub genes

**FIGURE 7:**
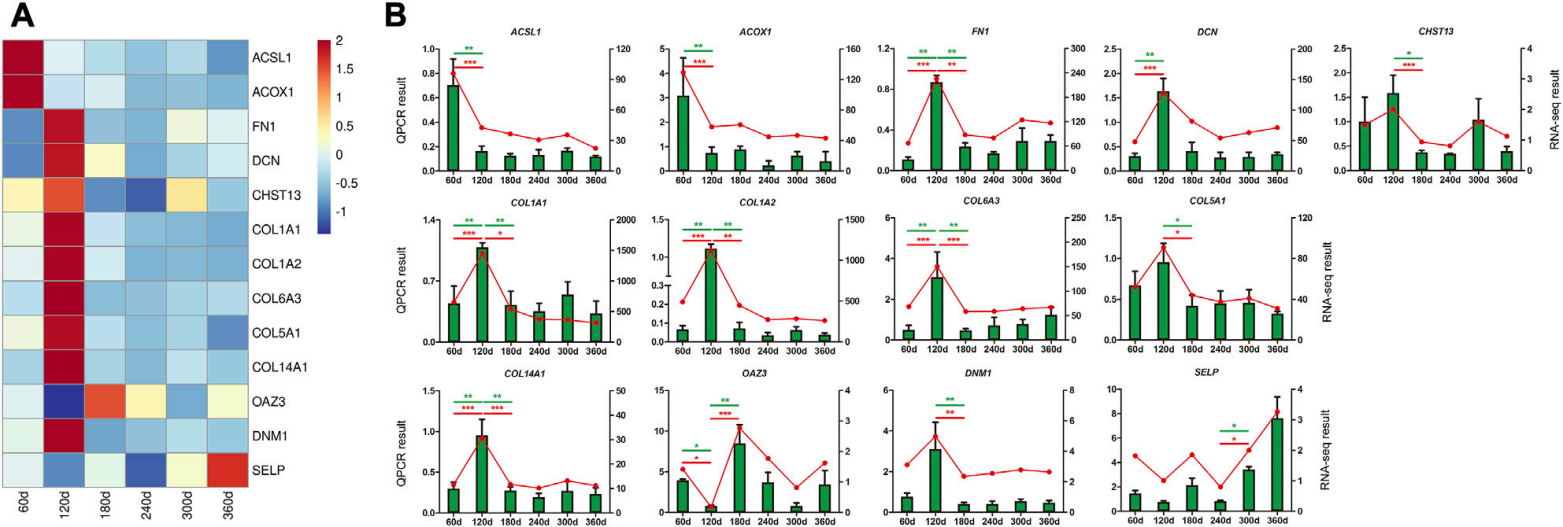
QPCR validation of DEGs and hub genes in backfat tissue. **(A)** The heatmap of DEGs and hub genes. **(B)** QPCR validation of DEGs and hub genes. The X-axis represented the developmental stages of Ningxiang pigs. QPCR result was exhibited as green column labeled on the Y-axis on the left, and *β-actin* was used as endogenous control. The data from RNA-seq result was displayed as red line labeled on the Y-axis on the right, and the expression was normalized as FPKM. Statistical analysis of the data from QPCR assay were performed by one-way analysis of variance program with SPSS 20.0 software, and the results were expressed as mean values and standard error. Differences were considered to be significant at *p* ≤ 0.05 (* *p* ≤ 0.05 and ** *p* ≤ 0.01).

Thirteen DEGs and hub genes from 8 modules were selected for validation by QPCR analysis, including *ACSL1*, *ACOX1*, *FN1*, *DCN*, *CHST13*, *COL1A1*, *COL1A2*, *COL6A3*, *COL5A1*, *COL14A1*, *OAZ3*, *DNM1*, and *SELP*. The expression trend of all these genes showed strong consistency with RNA-seq data (Figure 7), manifesting the reliability and accuracy of our study.

## Discussion

This study aimed at identifying 1,024 DEGs across five comparison groups during backfat tissue development in Ningxiang pigs. The WGCNA results depicted that six and three modules were predominantly associated with developmental stages and traits (backfat thickness, adipocyte area, and adipocyte number), respectively, and thirteen DEGs and hub genes were recognized for the first time.

The turquoise module was remarkably associated with the 60 days developmental stage. Enrichment analysis of this module revealed the significance of Acyl-CoA synthetase long chain family member 1 (*ACSL1*) and Acyl-CoA oxidase 1 (*ACOX1*), which are involved in the peroxisome, PPAR signaling pathway, fatty acid degradation, and biosynthesis of unsaturated fatty acids in the early developmental stage of backfat tissue. ACSL1 plays a crucial role in lipid metabolism; it converts long chain fatty acids to fatty acyl-CoAs by esterification, and *ACSL1* level is prominently elevated during the early stage of porcine preadipocyte differentiation (Shan et al., 2022). Additionally, *ACSL1* overexpression suppresses lipolysis and fatty acid β-oxidation and upregulates polyunsaturated fatty acid synthesis, triglyceride accumulation, and lipid droplet aggregation (Li et al., 2020a; Zhao et al., 2020; Shan et al., 2022). ACOX1, the first rate-limiting enzyme involved in peroxisomal fatty acid β-oxidation, is predominantly associated with lipid homeostasis and preadipocyte adipogenesis. Prior research depicted that hepatic *ACOX1* deficiency substantially lowers triglyceride accumulation in mice (He et al., 2020), and gain-of-function and loss-of-function assays demonstrated that *ACOX1*, governed by transcription factors C/EBPα and miR-25-3p, stimulates the adipogenesis of bovine intramuscular preadipocyte (Zhang et al., 2021). However, a recent study documented that *ACOX1* inhibition promotes triglyceride accumulation in mouse hepatocytes (Yang et al., 2023). The discrepancy in these findings may be attributed to specific experimental conditions and different cell sources. The present study exhibited considerably higher levels of *ACSL1* and *ACOX1* expression in 60 days, implying a conceivable action in the early developmental stage of backfat tissue in Ningxiang pigs.

Analysis of the red module, which was distinctly related to the 120 days developmental stage, revealed enrichment of the PI3K-Akt signaling pathway, extracellular matrix (ECM)-receptor interaction, protein digestion and absorption, and focal adhesion. *FN1*, *DCN*, *CHST13*, *COL1A1*, *COL1A2*, *COL5A1*, *COL6A3*, and *COL14A1* were further selected as the DEGs and hub genes. Intriguingly, these genes displayed similar expression trends in expression in backfat tissue across the six developmental stages, with the highest expression observed in 120 days.

Fibronectin (*FN*), one of the major fibrillary components of the ECM and an adipocyte-specific dysregulated gene product in obese adipose tissue, plays a vital role in tissue development, cell morphology, and mesenchymal stem cell differentiation (Wang et al., 2019; Yu et al., 2022). *FN1* knockout adversely affects the adipogenic differentiation of induced human infrapatellar fat pad-derived stem cells, as evidenced by remarkably low adipogenic gene expressions and lipid droplets (Wang et al., 2019). Our findings were corroborated by KEGG and protein-protein interaction network analysis, which presented *FN1* as a hub gene in subcutaneous adipose tissue derived adipocyte of obese patients; consequently, *FN1* and its associated signaling pathways could be rendered potential targets in treating obesity (Yu et al., 2022). Decorin (DCN), a small leucine rich proteoglycan component of the ECM in various tissues, is overexpressed in adipose tissue, binds to a variety of collagens, and contributes to collagen fibril formation (Meissburger et al., 2016; Svärd et al., 2019). The expression of glycanation site deficient *DCN* in 3T3-L1 cells promotes proliferation but suppresses lipid accumulation upon adipogenic induction (Daquinag et al., 2011). Another study suggested that the prevalence of *DCN* in murine visceral preadipocyte correlates with the reduced propensity of these cells to undergo adipogenic differentiation (Meissburger et al., 2016). Considering the essential functions of fibronectin and different types of collagen in adipocyte differentiation, the detrimental impact of *DCN* on adipogenesis may at least be partly attributable to alterations in ECM formation. These observations indicated a crucial role for *DCN* in adipogenesis. Carbohydrate sulfotransferase 13 (*CHST13*), a member of the carbohydrate sulfotransferase gene family, encodes a chondroitin sulfating and chondroitin sulfate synthesizing enzyme. Previous researches suggested that *CHST13* is prominently associated with several biological processes, including liver injury, cell invasion, and cancer (Ryanto et al., 2020; Wang et al., 2022; Southekal et al., 2023); however, the correlation between *CHST13* and adipose deposition remains largely unexplored and warrants further research.

Collagens expressed by adipose tissue, namely COL1A1, COL1A2, COL5A1, COL6A3, and COL14A1 (Ullah et al., 2013; Côté et al., 2017; Yu et al., 2022), are key constituents of the ECM and play crucial roles in regulating stem cell stemness, preadipocyte differentiation and adipose tissue expandability (Berger et al., 2015; Wang et al., 2019; Johnston and Abbott, 2022).

*COL1A1* is a major ECM gene in adipose tissue that is overexpressed following weight loss or reduction in obese adipose tissue (Berger et al., 2015). *COL1A2* is an adipogenic marker in multipotent antler stem cells, and any drastic change in distinct adipocyte morphology and accumulated lipid droplets is paralleled by a 2.5-fold upregulation of *COL1A2* expression (Berg et al., 2007). Additionally, *COL1A1* and *COL1A2* have been identified as hub genes in obesity-induced cardiac fibrosis (Pan et al., 2022). *COL5A1* is differentially expressed before and after bariatric surgery and may be a novel candidate gene for modulating adipose tissue function (Dankel et al., 2010). In *PLXND1* gene deficient zebrafish visceral adipose tissue, the induction of *COL5A1* promotes adipocyte proliferation and differentiation, culminating in hyperplastic visceral adipose tissue morphology and reduced lipid accumulation (Minchin et al., 2015). Moreover, *COL5A1* has been deemed a hub gene associated with bovine subcutaneous adipose tissue by WGCNA, which is consistent with our results (Sheng et al., 2022). Increasing evidence suggests that *COL6A3* expression is distinctly correlated with adipose tissue mass, adipocyte size, weight gain or loss, insulin resistance, and inflammation (Pasarica et al., 2009; Dankel et al., 2014; McCulloch et al., 2015). *COL6A3* knockdown elevates the expression of adipogenic genes and triglyceride content in human adipocyte (Gesta et al., 2016). *COL14* is a fibril-associated collagen that is predominantly expressed in well differentiated tissues, and it could potentially trigger the differentiation of 3T3-L1 preadipocyte into adipocyte, as evidenced by lipid accumulation (Ruehl et al., 2005). Bioinformatic analysis revealed that *COL14A1* is downregulated in the ECM of adipogenically differentiated mesenchymal stem cells (Ullah et al., 2013), indicating its significance in adipose tissue development.

Recent investigations have verified that the COL1 network is the last to form and remains well organized during the later stage of adipocyte differentiation, and the extracellular network of COL5 and COL6 is formed in the middle stage of adipocyte differentiation and maintained until the late stage of adipocyte differentiation (Sheng et al., 2022). Another study has designated *COL1A2*, *COL5A1*, *COL6A3* as DEGs in obese and healthy adipocyte excised from subcutaneous tissue in humans. GO analysis demonstrated that these three DEGs are enriched in ECM organization in biological process; in ECM and collagen trimer in cellular component. KEGG pathway enrichment further revealed that the aforementioned three DEGs are primarily involved in ECM-receptor interaction, protein digestion and absorption, and PI3K-Akt signaling pathway (Yu et al., 2022). These results are in accordance with those of our investigation, although the functional relevance of *COL1A1*, *COL1A2*, *COL5A1*, *COL6A3*, and adipogenesis needs to be further elucidated.

The pink, paleturquoise, darkorange, and darkgreen module had the highest correlation coefficient with 180, 240, 300, and 360 days developmental stage, respectively. Besides, tan, black and turquoise module had strong relationship with backfat thickness, adipocyte area and adipocyte number in turn. *OAZ3*, *SEL*P, and *DNM1* were apprehended as DEGs and hub genes in the pink, tan, and black module, respectively. Ornithine decarboxylase antizyme 3 (*OAZ3*) is a member of the antizyme gene family, and its mRNA is exclusively expressed in post-meiotic male germ cells (Ruan et al., 2011). *OAZ3* evidently aids in the regulation of polyamine concentration during spermiogenesis and contributes to sperm function and fertility (Gòdia et al., 2020; Sarkar et al., 2022). P-selectin (*SELP*) belongs to the selectin proteins family and is primarily expressed in platelets, endothelial cells, and immune cells (Wang et al., 2023). *SELP* modulates thrombus formation through platelets activation, and a positive correlation has been observed between age and *SELP* expression in hyperlipidemia and thrombosis related diseases (Koyama et al., 2003; Yeini and Satchi-Fainaro, 2022). Nevertheless, few investigations have reported the relationship between *OAZ3*, *SELP* and adipogenic differentiation and adipogenesis, to date. This study provides novel mechanistic insights into the regulation of adipogenesis. Dynamin 1 (DNM1), a member of the dynamin superfamily of proteins, plays a central role in mitochondrial and peroxisomal distribution and fission processes (Koch et al., 2003; Tamura et al., 2011) and has displayed functionality in brown and white adipose tissue. It has been reported that the lipid droplets in adipose tissue of adipose tissue-specific *DNM1* knockout mice exhibit more unilocular morphology with larger sizes, with *DNM1* deficiency effecting abnormal retention of nascent micro-lipid droplets in endoplasmic reticulum, decreased lipolysis, and accumulation of unhealthy adipocyte in adipose tissue. In contrast, the retention of lipid droplets in endoplasmic reticulum can be rescued by *DNM1* overexpression in adipocyte (Li. et al., 2020). Furthermore, *DNM1* is overexpressed in brown adipose tissue, and its level escalates during beige and brown adipocyte differentiation. Inhibition of *DNM1* expression was confirmed to mitigate beige adipocyte differentiation, thereby validating its essential role in beige and brown adipogenesis (Mooli et al., 2020).

## Conclusion

**FIGURE 8:**
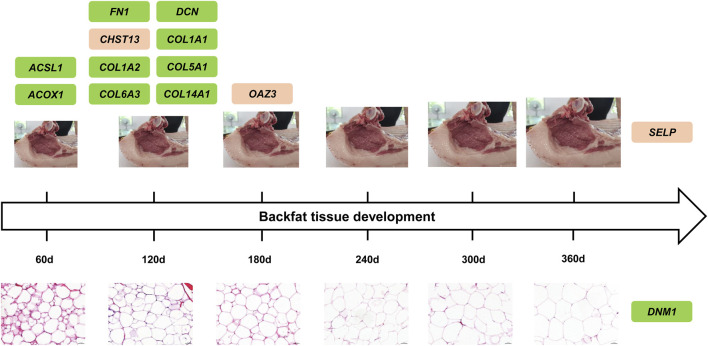
Proposed model of DEGs and hub genes during developmental stage of backfat tissue in Ningxiang pigs. The upper row of figures represented the developmental trend of backfat tissue, and the size of figures exemplified backfat thickness. The lower row of figures manifested adipocyte area in backfat tissue. Additionally, the green rectangles represented genes are closely associated with adipogenesis and lipid metabolism which have been validated previously, while the orange rectangles represented genes might involve in adipogenesis and lipid metabolism which warrants further investigation.

Taken together, a total of thirteen DEGs and hub genes were recognized from six developmental stages related modules and three trait related modules in backfat tissue in Ningxiang pigs, among which, *ACSL1* and *ACOX1*, well known biomarkers of adipogenesis, might perform function in the early developmental stage of backfat tissue (60 days). Other DEGs and hub genes in modules, such as *FN1*, *DCN*, *COL1A1*, *COL1A2*, *COL5A1*, *COL6A3*, and *COL14A1*, play regulatory roles in cell adipogenic differentiation, lipid droplet accumulation, triglyceride content, and adipose tissue mass, illustrating their unignorable position around 120 days developmental stage of backfat tissue in Ningxiang pigs (Figure 8). In addition, *OAZ3*, *SELP* and *DNM1* were also identified as hub genes, and the functional relevance associated with the lipid metabolism requires further elucidation.

## Data Availability

The data presented in the study are deposited in the NCBI repository, accession number GSE234796.

## Supplementary material

SUPPLEMENTARY TABLE S1 The sequences of primers in QPCR analysis.

SUPPLEMENTARY TABLE S2 Statistical data of RNA-seq.

SUPPLEMENTARY TABLE S3 The detailed information about DEGs in five comparison groups.

SUPPLEMENTARY TABLE S4 The detailed information about modules.

SUPPLEMENTARY TABLE S5 GO analysis for the genes in developmental stage related and trait related modules.

SUPPLEMENTARY TABLE S6 KEGG enriched pathways for the genes in developmental stage related modules.

SUPPLEMENTARY TABLE S7 KEGG enriched pathways for the genes in trait related modules.

SUPPLEMENTARY TABLE S8 The connectivity between genes in developmental stage related and trait related modules.

SUPPLEMENTARY FIGURE S1 The top 20 significantly GO terms in developmental stage related and trait related modules. **(A)** Turquoise module. **(B)** Red module. **(C)** Pink module. **(D)** Paleturquoise module. **(E)** Darkorange module. **(F)** Darkgreen module. **(G)** Tan module. **(H)** Black module.

## Abbreviations

*ACSL1*, acyl-CoA synthetase long chain family member 1; *ACOX1*, acyl-CoA oxidase 1; *CHST13*, carbohydrate sulfotransferase 13; *COL*, collagen; *DCN*, decorin; DEGs, differentially expressed genes; *DNM1*, Dynamin 1; ECM, extracellular matrix; *FN1*, fibronectin 1; FPKM, fragments per kilobase of transcript per million; GO, gene ontology; HE, hematoxylin and eosin; KEGG, kyoto encyclopedia of genes and genomes; *OAZ3*, ornithine decarboxylase antizyme 3; QPCR, quantitative real-time polymerase chain reaction; RNA-seq, RNA-sequencing; *SELP*, P-selectin; WGCNA, weighted gene co-expression network analysis.
