# Supplementary material for: Identification of crucial modules and genes associated with backfat tissue development by WGCNA in Ningxiang pigs
Source: Front Genet. 2023 Aug 17;14:1234757. doi: 10.3389/fgene.2023.1234757 (PMC10469685; doi:10.3389/fgene.2023.1234757)
Supplement: Supplementary file 4 [file Table1.DOC]

Supplementary Table S1 The sequences of primers in QPCR analysis.

| Gene |  | Primer sequence |
| --- | --- | --- |
| *ACSL1* | Forward | CGGAAACCAGACCAACCCTA |
|  | Reverse | TGAGCGAAGATGCCGACGAA |
| *ACOX1* | Forward | GAAGAAGATAAGGGAGTTTGGC |
|  | Reverse | ACCCATCTCTGTCTGAGCATAA |
| *FN1* | Forward | CACTCAGGTAACACCCACAAG |
|  | Reverse | GATGCTCACTTCATACTTGG |
| *DCN* | Forward | ATCGTCGTAGAACTTGGCAC |
|  | Reverse | GATGGTGGTAATGTTGGTGTCAGC |
| *CHST13* | Forward | CAAATAATGGGGTCTCGGCTGG |
|  | Reverse | CCCAATCCACCCACATCACG |
| *COL1A1* | Forward | CGGGTTCGGAGGAAAGTCAGGA |
|  | Reverse | AAAATGAGGAGCCCCTGCCACC |
| *COL1A2* | Forward | GGCTCTGCTACACAAGGAGTCT |
|  | Reverse | GCATGTTGCTAGGCACGAAGT |
| *COL6A3* | Forward | GTATCCTTCCGCTTGGTTCAT |
|  | Reverse | AGTTGGCTTCTGCCTCCCTA |
| *COL5A1* | Forward | ACCCGCTCTTCAGAGGCATCAACC |
|  | Reverse | GCTGTAGTGCTCACAGTAATCGTA |
| *COL14A1* | Forward | CAACTTCAGACTGGTTCGGT |
|  | Reverse | CCCTTGTATGGTAGATTTCG |
| *OAZ3* | Forward | CATACGCCTATTATCTTTAC |
|  | Reverse | GAGTGTCAAGTCCTCCTGTTCT |
| *SELP* | Forward | GCTAAGAATGAGCCCAATAACG |
|  | Reverse | AGCCCGCTTTCTTTTCACACAA |
| *DNM1* | Forward | GATGGACGAAGGAACAGATGC |
|  | Reverse | GGTTTGTCAGTTGCTGGTTGAG |
| *β-actin* | Forward | GACCCAGATCATGTTCGAGACCT |
|  | Reverse | CGGAGTCCATCACGATGCCAG |
