# Supplementary material for: Identification of crucial modules and genes associated with backfat tissue development by WGCNA in Ningxiang pigs
Source: Front Genet. 2023 Aug 17;14:1234757. doi: 10.3389/fgene.2023.1234757 (PMC10469685; doi:10.3389/fgene.2023.1234757)
Supplement: Supplementary file 5 [file Table2.DOCX]

Supplementary Table S2 Statistical data of RNA-seq.

| Sample | Raw data | | Valid data | | Valid ratio(%) | Q20(%) | Q30(%) | GC content(%) | Mapped reads | Unique Mapped reads | Expressed transcripts | Expressed genes |
| --- | --- | --- | --- | --- | --- | --- | --- | --- | --- | --- | --- | --- |
|  | Read | Base | Read | Base |  |  |  |  |  |  |  |  |
| 60d1 | 89351696 | 13.40G | 81225086 | 12.18G | 90.90 | 99.96 | 97.65 | 45.50 | 74335200(91.52%) | 72956982(89.82%) | 56691 | 23761 |
| 60d2 | 92919158 | 13.94G | 84973004 | 12.75G | 91.45 | 99.96 | 97.69 | 46.00 | 77749360(91.50%) | 76203165(89.68%) | 57244 | 23916 |
| 60d3 | 93323340 | 14.00G | 84786554 | 12.72G | 90.85 | 99.95 | 97.68 | 46.50 | 77357881(91.24%) | 75768320(89.36%) | 56294 | 23628 |
| 120d1 | 91151424 | 13.67G | 83955630 | 12.59G | 92.11 | 99.95 | 97.89 | 47.50 | 76480234(91.10%) | 74703149(88.98%) | 57337 | 23976 |
| 120d2 | 97751248 | 14.66G | 90086352 | 13.51G | 92.16 | 99.95 | 97.99 | 47.50 | 82283965(91.34%) | 80326910(89.17%) | 57783 | 24057 |
| 120d3 | 91659732 | 13.75G | 83795302 | 12.57G | 91.42 | 99.95 | 97.93 | 46.50 | 76647756(91.47%) | 74780958(89.24%) | 56858 | 23636 |
| 180d1 | 95043110 | 14.26G | 86238066 | 12.94G | 90.74 | 99.95 | 97.76 | 47.00 | 78935258(91.53%) | 77116795(89.42%) | 57006 | 23761 |
| 180d2 | 91690486 | 13.75G | 83596190 | 12.54G | 91.17 | 99.96 | 97.77 | 46.00 | 76786444(91.85%) | 75063895(89.79%) | 56367 | 23416 |
| 180d3 | 92403018 | 13.86G | 84613464 | 12.69G | 91.57 | 99.95 | 97.76 | 46.50 | 76946333(90.94%) | 74645416(88.22%) | 56585 | 23554 |
| 240d1 | 90652220 | 13.60G | 83372548 | 12.51G | 91.97 | 99.95 | 97.94 | 47.00 | 76057610(91.23%) | 73804504(88.52%) | 56892 | 23753 |
| 240d2 | 90212794 | 13.53G | 82347188 | 12.35G | 91.28 | 99.94 | 97.76 | 47.50 | 74447013(90.41%) | 71910001(87.33%) | 56820 | 23658 |
| 240d3 | 91232866 | 13.68G | 84031426 | 12.60G | 92.11 | 99.96 | 97.97 | 46.50 | 76702757(91.28%) | 74905255(89.14%) | 56602 | 23568 |
| 300d1 | 91538426 | 13.73G | 83118524 | 12.47G | 90.80 | 99.95 | 97.72 | 47.00 | 75476239(90.81%) | 73250769(88.13%) | 56466 | 23528 |
| 300d2 | 96264794 | 14.44G | 89120386 | 13.37G | 92.58 | 99.95 | 97.93 | 47.50 | 80923663(90.80%) | 79035093(88.68%) | 57314 | 23720 |
| 300d3 | 95111474 | 14.27G | 87435340 | 13.12G | 91.93 | 99.95 | 97.71 | 47.50 | 79199045(90.58%) | 76883680(87.93%) | 56757 | 23755 |
| 360d1 | 89249470 | 13.39G | 82657458 | 12.40G | 92.61 | 99.95 | 97.93 | 47.50 | 75165297(90.94%) | 73227941(88.59%) | 56472 | 23573 |
| 360d2 | 97502936 | 14.63G | 89642168 | 13.45G | 91.94 | 99.95 | 97.70 | 46.50 | 81619567(91.05%) | 79654508(88.86%) | 56901 | 23650 |
| 360d3 | 93339178 | 14.00G | 85993544 | 12.90G | 92.13 | 99.92 | 97.56 | 51.50 | 75115005(87.35%) | 69614057(80.95%) | 56492 | 23806 |
